# Wildfire smoke impacts activity and energetics of wild Bornean orangutans

**DOI:** 10.1038/s41598-018-25847-1

**Published:** 2018-05-15

**Authors:** W. M. Erb, E. J. Barrow, A. N. Hofner, S. S. Utami-Atmoko, E. R. Vogel

**Affiliations:** 1 Department of Anthropology, Rutgers University, New Brunswick, NJ USA; 2 Center for Human Evolutionary Studies, Rutgers University, New Brunswick, NJ USA; 3 CORE Borneo, New Brunswick, NJ USA; 4 Fakultas Biologi, Universitas Nasional, Jakarta, Indonesia; 5 Primate Research Centre, Universitas Nasional, Jakarta, Indonesia

## Abstract

Indonesia’s peatlands experience frequent and intense wildfires, producing hazardous smoke with consequences for human health, yet there is a lack of research into adverse effects on wildlife. We evaluated the effects of smoke on the activity and energy balance of Bornean orangutans (*Pongo pygmaeus wurmbii*) in a peat swamp forest at the Tuanan Research Station, Central Kalimantan. We collected behavioural data and urine samples from four adult flanged males before, during, and after wildfires between March 2015 and January 2016. During fires, particulate matter (PM_10_) concentrations were hazardous. Orangutans increased rest time during and after the smoke period, and decreased travel time and distance and increased fat catabolism post-smoke. The increase in post-smoke ketones was not related to changes in caloric intake and was likely due to an increase in energy expenditure, possibly related to immune response. Results show that wildfire smoke negatively affects orangutan condition, and sustained research is needed to assess the magnitude of the threat to the long-term viability of this Critically Endangered species.

## Introduction

Wildfires have occurred in Southeast Asia for millennia, but are increasingly frequent and intense, with the most extensive fires in Indonesia’s peatlands^1^. Peat fires are a perfect storm resulting from a combination of human activities during recent decades that have dramatically altered these ecosystems via deforestation and draining for conversion to agricultural lands^2^. Repeated burnings have made these landscapes more susceptible to fires, exacerbated by drought conditions produced by strong El Niño weather systems^3^. Peatland fires not only destroy thousands of hectares of forest annually, but also release tons of carbon and other gases, contributing to global climate change, and producing hazardous air pollution^4^. The most disastrous of Indonesia’s fires occurred in 1997 and contributed the equivalent of as much as 40% of the average annual global carbon emissions from the burning of fossil fuels^3^.

In addition to greenhouse gases, wildfire smoke contains numerous hazardous components, including particulate matter (PM), the leading cause of worldwide pollution-related mortality, and carbon monoxide (CO), a poisonous gas that reduces the amount of oxygen transported to critical organs, including the heart and brain^4^. In 2015, Indonesia’s peat fires exhibited the most severe fire activity and smoke pollution recorded since NASA’s Earth Observing satellite system began observations in the early 2000s^5^. The widespread haze produced from these peat fires had significant impacts on human health, causing an estimated 100,300 deaths in the aftermath of the fire^6^. Most of these deaths likely occurred over the course of the first year after the fire (Loretta Mickley, personal communication).

Wildfire smoke appears to affect the health of nonhuman animals as well, causing eye and throat irritation, breathing difficulty, and impaired immune function in livestock animals like sheep and horses^7^. Furthermore, a study of Bornean gibbons (*Hylobates albibarbis*) during the 2006 peat fires found that individuals sang fewer and shorter songs on smoky days^8^. Given the consequences of haze for human health, these behavioural changes could indicate underlying health effects, though we are not aware of any studies documenting adverse health effects from forest fire smoke for wildlife.

Borneo’s peatlands contain a large proportion of the island’s fauna and provide critical habitats for many threatened species, including the Critically Endangered Bornean orangutan (*Pongo pygmaeus*)^9,10^. Peat-dwelling orangutans are ideal models to investigate how smoke affects wildlife health, as they are an indicator species whose health and behaviour signal environmental quality^11^. For instance, when fruit is scarce, Bornean orangutans conserve energy by decreasing their active period, travel time, and travel distance, and metabolize fat reserves to produce energy^12–15^, and in disturbed habitats, travel shorter distances and spend more time resting^16,17^.

There is a paucity of research into the behavioural changes of animals surviving wildfires, with a noteworthy exception: antechinus (*Antechinus stuartii*), small insectivorous marsupials, decrease daytime activity and increase energy-conserving torpor use following both prescribed fires and wildfires in Australia. These shifts are thought to be key to individuals’ survival by reducing their energy requirements in burnt areas where food resources are severely reduced, and have been proposed to provide a competitive edge to heterothermic mammals that are able to adjust their energy requirements^18,19^. While this research provides fascinating insights into the behavioural responses of small insectivorous mammals in burned habitats, no studies have documented similar energy conservation strategies by animals in unburned areas in response to smoke exposure.

Indonesia’s 2015 peat fires provided a unique opportunity to document, for the first time, changes in orangutan behaviour and health in response to wildfire smoke. As a leading cause of haze and the air pollutant most strongly linked to adverse health effects in humans – including asthma, heart attack, and early death^20^ – we predicted that high particulate matter (PM_10_) would negatively impact orangutan energetic condition, reflected in reduced travel times, active periods, and travel distances, and increased resting times and ketosis.

## Methods

### Study Site and Subjects

**Figure 1:**
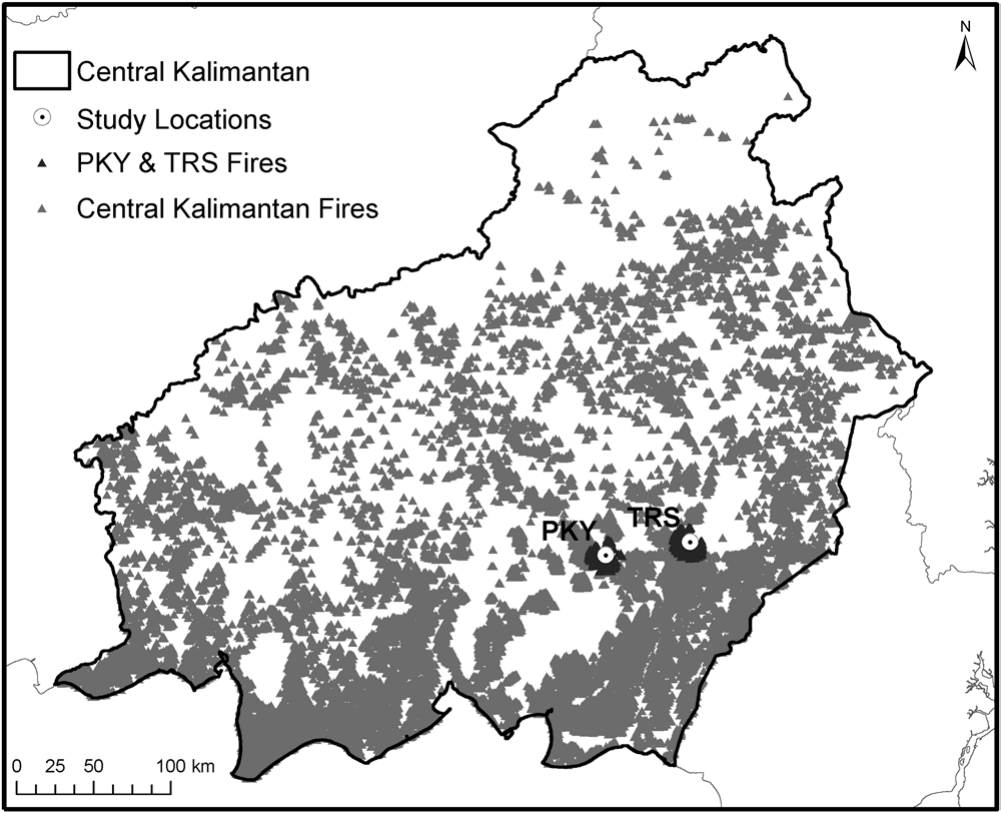
Map depicting 2015 fire hotspots (grey triangles) across Central Kalimantan detected by satellite (MODIS Active Fire Detections data set), and those within 10 km of Tuanan (TRS) and Palangkaraya (PKY) study locations (black triangles). Map created using ESRI® ArcMap^TM^ 9.3 http://www.esri.com/software/arcgis/eval-help/arcgis-93.

We conducted research at the Tuanan Orangutan Research Station situated within a 105,166 ha protected area (KPHL Model Kapuas) in Central Kalimantan, Indonesia (2°09′06.1′′S; 114°26′26.3′′E, Fig. 1). Tuanan lies within the Ex-Mega Rice Project, a million-hectare agricultural land conversion project implemented in 1996–1997 that resulted in the construction of more than 4,500 km of drainage and irrigation channels. As a result, more than half of this area burned during the El Niño-driven wildfires of 1997–1998, contributing to a loss of 72% of peat swamp forests in the following nine years and leaving much of the peatland deeply degraded^4,21^. The study area comprises approximately 900 hectares of regenerating peat swamp forest, about 10% of which burned in 2015. Although Tuanan exhibits less pronounced fluctuations compared to the dipterocarp-dominated masting forests of Borneo, the availability of fruit demonstrates strong inter-annual variation in the magnitude and timing of peak-fruit periods^22^.

Data were collected between March 2015 and January 2016 as part of a long-term investigation into flanged male orangutan energetics initiated in 2013. We divided the data into three periods: pre-smoke (March–July 2015), smoke (September–October 2015), and post-smoke (November 2015–January 2016). These periods were delineated by the smoke conditions at the research station, recorded daily as the presence or absence of visible smoke by research station staff. WME, EJB, ANH, and local research assistants conducted full-day observations of four habituated adult flanged males across the three study periods (pre-smoke: N = 18 days, mean = 4.5 days/male; smoke N = 20 days, mean = 5 days/male; post-smoke N = 16 days, mean = 4 days/male; Supplementary Table S1).

### Data and Sample Collection

We collected behavioural data during nest-to-nest focal animal follows, using two-minute instantaneous sampling to record activity and feeding^23^. We defined active period as the duration from the first activity point after the focal animal left the nest in the morning until the first activity point when the focal was resting in its nest for the evening. Rest time and travel time were calculated as the proportion of instantaneous samples during the active period when the focal animal was resting or travelling, respectively^24^. For feeding bouts, we recorded the species, part, and ripeness of food items; calculated energy intake per feeding bout using item-specific feeding rates and nutritional content; and summed all feeding bouts to estimate daily caloric intake (following the nutritional methods and energetic calculations reported in^15,22^). Ranging data were collected using a handheld GPS unit (Garmin GPSMap 62s), which we used to record the location of the focal animal at 10-minute intervals, and we calculated daily path lengths using the HoRAE toolbox in OpenJUMP^25^.

To determine whether animals were in ketosis, we collected urine samples opportunistically, prioritising the first urination each morning (modal collection time = 05:00). Samples were collected by positioning a plastic bag under the urinating animal, or by immediately pipetting urine from the leaves or ground. We did not collect samples contaminated with faeces, and used Chemstrip® Test Strips (10 SG or UA, Roche Diagnostics USA) to test for ketones as soon as possible (mean elapsed time = 6.1 minutes).

To document ecological habitat changes, we monitored all of the trees in three phenological plots containing ~1,650 tagged trees (diameter at breast height >10 cm) covering 2.3 ha. We calculated the monthly fruit availability index (FAI) as the number of trees bearing fruits divided by the total number of trees sampled^15,26^.

We acquired archived data on fire hotspots in Central Kalimantan from the NASA Fire Information for Resource Management System (FIRMS) MODIS active fire data set^27^. Air quality was assessed via concentrations of particulate matter (PM_10_), collected daily by the BMKG (Meteorology, Climatology & Geophysics Council) in Palangkaraya, located approximately 55 km west of Tuanan. In accordance with the Clean Air Act, the U.S. Environmental Protection Agency strictly regulates this pollutant, and PM_10_ values exceeding 150 µg/m^3^ in a 24-hour period are considered harmful to public health and the environment. Using the NASA FIRMS MODIS active fire data set, we quantified the number of fire hotspots within a 10-km radius of the research station as well as Palangkaraya, where the air quality data were collected.

### Data Analysis

For each dependent variable (daily travel time, rest time, active period, travel distance, ketones presence, and caloric intake), we used general linear mixed models (LMM) to determine: 1) immediate effects, i.e., those observed during the smoke period (pre-smoke vs. smoke) and 2) persistent effects, i.e., those observed after the smoke period (pre-smoke vs. post-smoke), including individual ID as a random effect and fruit availability (FAI) as a covariate. We conducted diagnostic tests of all models (residual distribution, leverage, and influence)^28^. Statistical analyses were performed using Statistica 13 (Dell Inc., Tulsa, OK). We used two-tailed tests, set alpha at 0.05, and corrected for multiple comparisons against the pre-smoke dataset following^29^.

### Ethical Approval

Research permits were issued by RISTEK-DIKTI (Permit #137/SIP/FRP/SM/V/2013) following the legal requirements of conducting research in Indonesia, and all research methods were approved by the Institutional Animal Care and Use Committee at Rutgers University (Protocol #11-030).

### Data Availability Statement

All biological data analysed for this study are included in this published article and its Supplementary Information file. The air quality data that support the findings of this study are available from BMKG (Meteorology, Climatology & Geophysics Council) in Palangkaraya but restrictions apply to the availability of these data, which were used under license for the current study, and so are not publicly available. Data are, however, available from the authors upon reasonable request and with permission of BMKG Palangkaraya.

## Results

### Fire Activity and Air Quality

**Figure 2:**
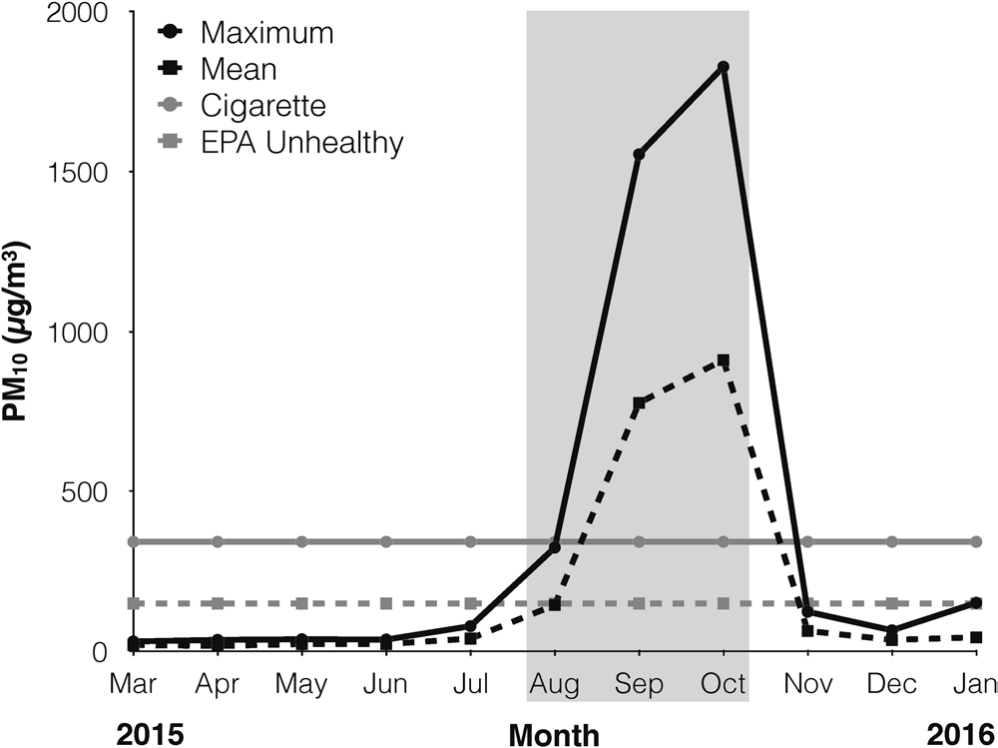
Maximum (solid black) and mean (dashed black) monthly PM_10_ values observed in Palangkaraya across the study period. PM_10_ after lighting a cigarette (solid grey)^38^ and the EPA unhealthy limit (dashed grey) for reference. Light grey rectangle indicates the smoke period.

Smoke conditions were recorded daily at the Tuanan Research station. We observed smoke from nearby forest fires beginning August 7 through November 6, 2015 (hereafter, fire season), when daily rains marked the arrival of the wet season. During the 2015 fire season, 79% of daily PM_10_ values recorded in Palangkaraya exceeded unhealthy levels (63 of 80 days sampled; mean = 588.0, range 61–1,829 µg/m^3^). In October, mean and maximum monthly values exceeded unhealthy levels by more than 6 and 12 times, respectively (Fig. 2).

**Figure 3:**
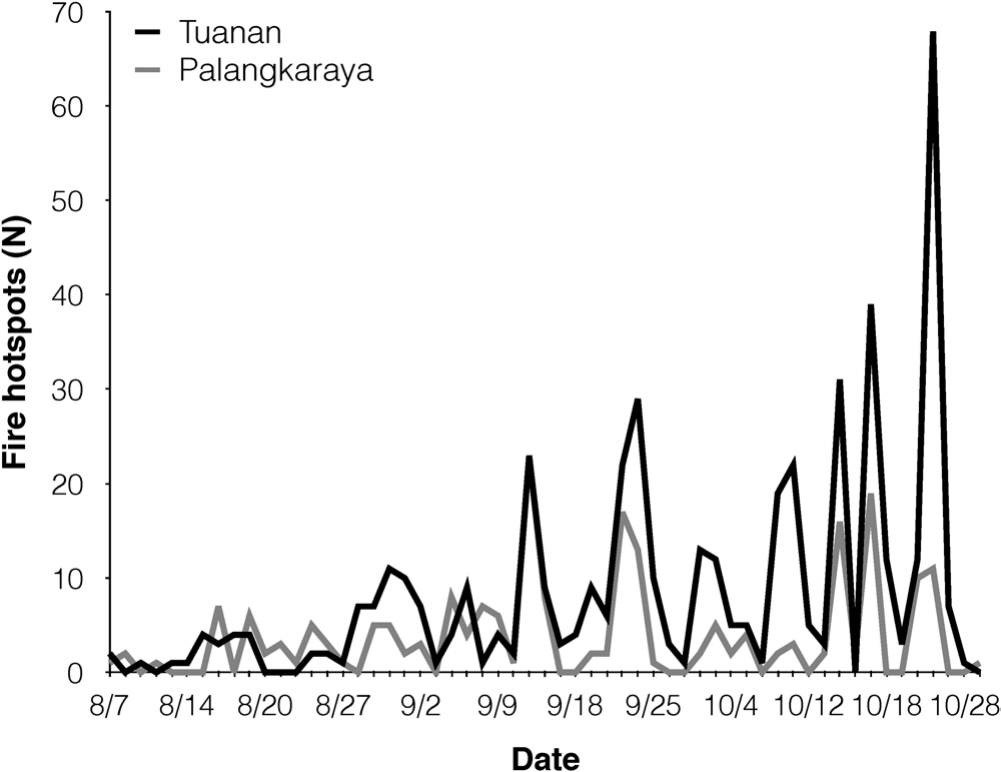
Daily fire hotspots detected by satellites (MODIS Active Fire Detections data set) at Tuanan (black) and Palangkaraya (grey) during the fire season.

During the fire season, a total of 218 fire hotspots (200 occurring on peatlands) were detected within 10 km of Palangkaraya (Fig. 3). At the same time, 465 hotspots (434 occurring on peatlands) were detected within 10 km of Tuanan (Fig. 3). Outside of the fire season, only eight hotspots (N = 7 pre-smoke, N = 1 post-smoke) were detected in Palangkara and five hotspots in Tuanan (pre-smoke only) in 2015. Daily fire activity at Tuanan (mean = 5.3, range 0–68 hotspots) was significantly greater than in Palangkaraya (mean = 2.5, range 0–23 hotspots; t = 3.45, p < 0.001) across the fire season.

We compared air quality across the three study periods during which we collected behavioural and ecological data at Tuanan (Pre-smoke: March 15–July 7, Smoke: September 4–October 17, Post-Smoke: November 19–January 13). Average daily air quality varied significantly across periods (F_2,149_ = 353.2, p < 0.0001). Pre- and post-smoke periods did not differ from each other (mean: pre-smoke = 22.2, post-smoke = 42.6 µg/m^3^; Tukey HSD: p = 0.77), but both had lower PM_10_ compared to the smoke period (mean: smoke = 830.2 µg/m^3^; Tukey HSD: p < 0.0001).

### Orangutan Activity and Energy Balance

**Figure 4:**
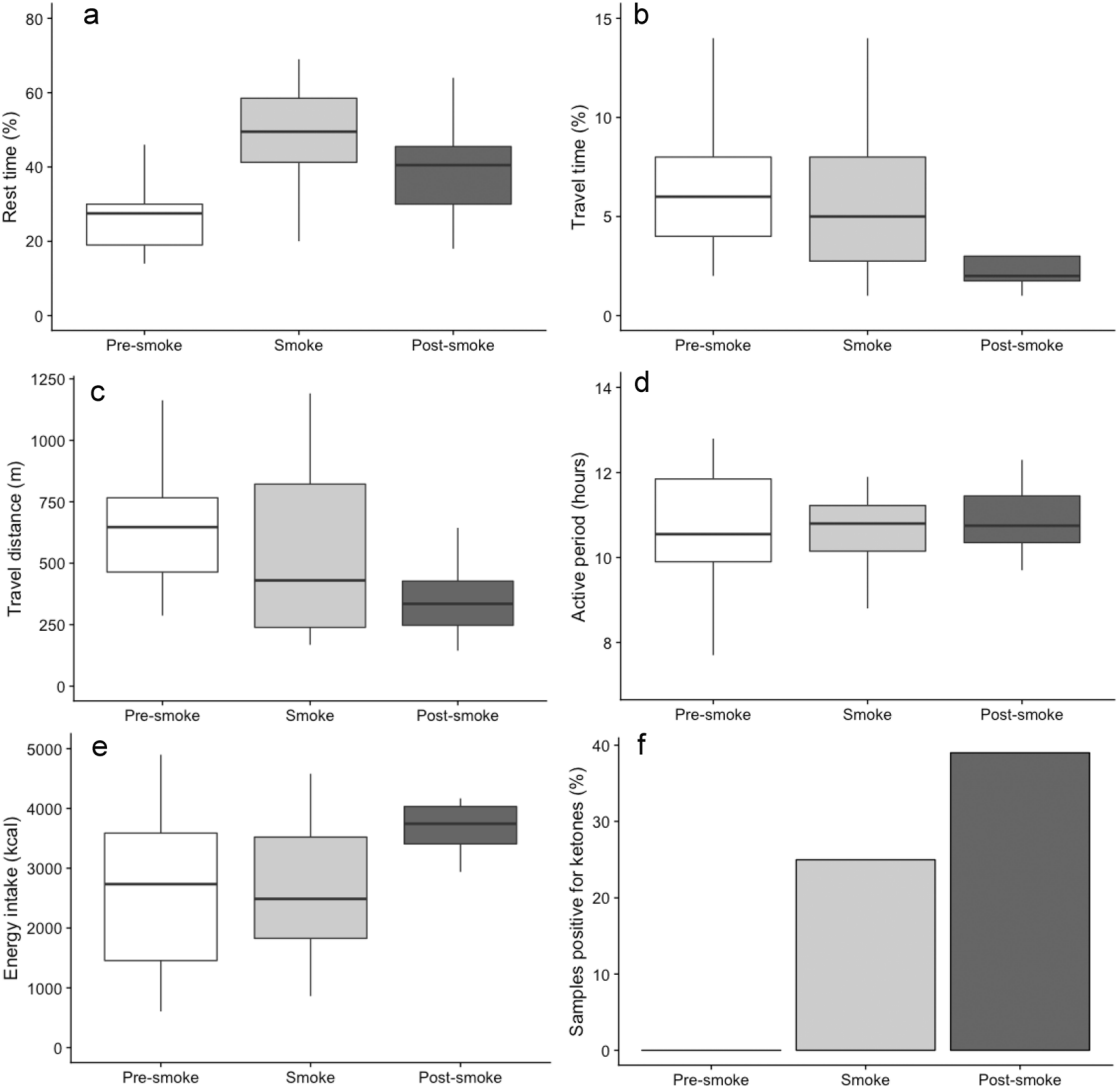
Box plots depicting orangutan activity and energetics across the three study periods (**a** = rest time, **b** = travel time, **c** = travel distance, **d** = active period, **e** = caloric intake, **f** = ketones).

We examined the immediate effects of smoke on orangutan behaviour by comparing data collected during the pre-smoke and smoke periods. Rest time increased significantly during the smoke period compared to baseline levels (Table 1a, Fig. 4a). Neither travel time, active period, nor travel distance significantly differed across these periods (Table 1a, Fig. 4b–d).

**Table 1:** LMM results for (a) immediate (smoke) and (b) persistent (post-smoke) changes in activity and energetics; *italics* = significant after Hochberg correction.

(a) Immediate	FAI		ID		Smoke	
Variable	F	p	F	p	F	p
*Rest time*	0.38	0.006	0.94	0.434	20.10	<*0*.*001*
Travel time	3.92	0.056	5.48	0.004	0.65	0.428
Travel distance	0.97	0.333	0.321	0.810	0.06	0.812
Active period	0.09	0.762	6.07	0.002	0.16	0.691
Calorie intake	0.33	0.568	3.69	0.022	0.17	0.679
Ketones	6.85	0.014	7.99	<0.001	0.54	0.466
**(b) Persistent**	**FAI**		**ID**		**Post-Smoke**	
*Rest time*	13.76	0.001	9.79	<0.001	10.92	*0*.*003*
*Travel time*	0.08	0.774	13.14	<0.001	16.75	<*0*.*001*
*Travel distance*	0.00	0.977	1.20	0.327	10.92	*0.003*
Active period	0.88	0.356	10.15	<0.001	4.26	0.048
*Calorie intake*	0.96	0.336	15.92	<0.001	84.18	*0.001*
*Ketones*	0.87	0.359	0.77	0.520	7.69	<*0.011*

We tested for possible persistent effects of smoke by comparing data collected during pre-smoke and post-smoke periods. As predicted, rest time increased and travel time and travel distance decreased (Table 1b, Fig. 4a–c). Following correction for multiple comparisons, active period did not change during the post-smoke period.

**Figure 5:**
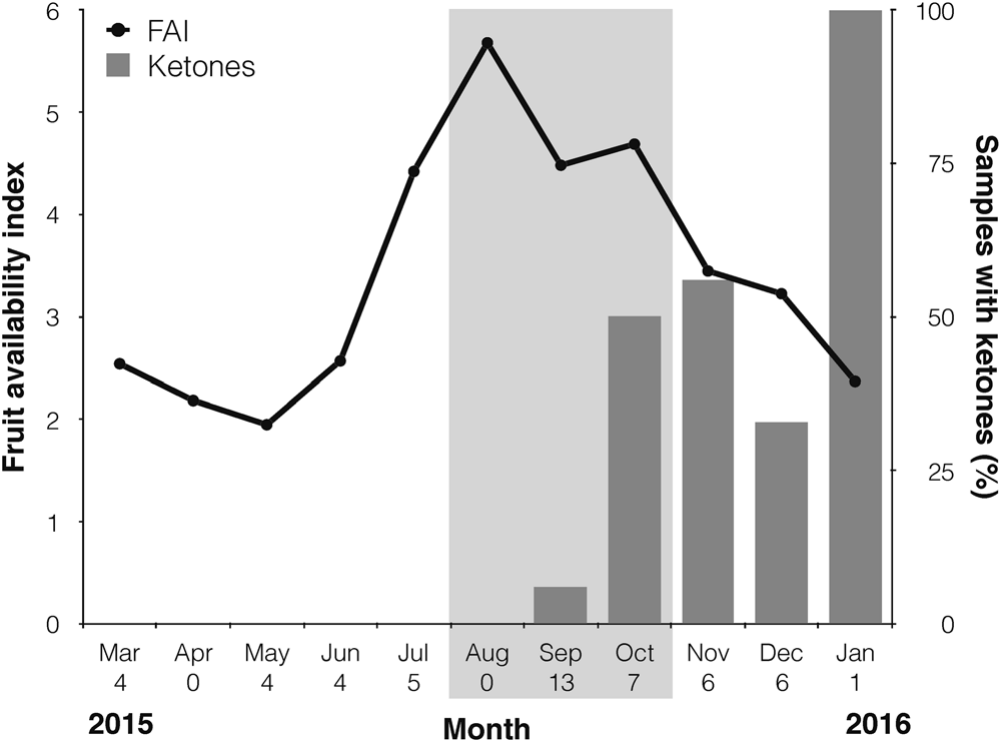
Urine samples testing positive for ketones (bars) in relation to fruit availability (line) across the study period. Monthly sample sizes are indicated on the x-axis. Light grey rectangle indicates the smoke period.

We analysed 60 urine samples for the presence of ketones across the three study periods (pre-smoke = 19, smoke = 24, post-smoke = 17 samples). Neither the proportion of samples with ketones nor energy intake differed between pre-smoke and smoke periods (Table 1, Fig. 4e,f). In contrast, both the proportion of samples with ketones and energy intake showed significant increases during the post-smoke period, indicating that the increase in ketone production was not due to reduced calorie consumption (Table 1, Fig. 4e,f). Although fruit availability reached its lowest in May, and was significantly lower during the pre-smoke period compared to the post-smoke period (pre-smoke average = 2.82, minimum = 1.95; post-smoke average = 3.26, minimum = 2.37; t = 19.6, p < 0.001), ketones were not detected during the pre-smoke period (Fig. 5).

## Discussion

Our study provides the first evidence that wildfire smoke affects orangutan condition. Orangutans reduced their energy budgets by resting more during and after smoke, and spent less time travelling shorter distances while experiencing negative energy balance post-smoke. These changes are similar to those observed during periods of fruit scarcity, when Bornean orangutans adopt an energy-conserving strategy^12,15^. In the Tuanan population, in particular, orangutans have been observed to fall into a negative energy balance state, as evidenced by significantly higher levels of ketone production, during periods of low fruit availability when energy intake is lowest^30^. As ketones indicate animals are catabolizing fat as an energy source, it appears orangutans in this study had entered a state of negative energy balance, expending more energy than they consumed post-smoke. Despite lower fruit availability and calorie consumption pre-smoke, no ketones were detected during that period, indicating that post-smoke ketosis was not merely due to reduced energy intake. It is thus more likely that orangutans had increased their energy expenditure post-smoke, despite energy-conserving behavioural changes in physical activity, namely resting and travelling. Such an increase in energy expenditure could occur if animals were experiencing allostatic load, for instance, through increased stress hormone production or immune response^31^. Interestingly, human subjects exposed to high PM_10_ levels during the 1997 forest fires showed elevated circulating cytokines (proinflammatory signalling proteins: IL-1β, IL-6, and GM-CSF), indicating a systemic inflammatory response, likely due to the adverse cardiopulmonary effects of particulate air pollution^32^. As systemic inflammatory responses can elevate energy expenditure and cause weight loss in humans^33^, increased inflammation presents a possible mechanism for the ketosis we observed post-smoke, and future studies should explore changes in cytokines and other relevant biomarkers (e.g., neopterin, cortisol) in response to wildfire smoke.

Overall, most of our predictions were supported, though our study design and the small number of individuals sampled limit our conclusions. Although for most variables, significant changes were only detected post-smoke, we cannot conclude that orangutans did not experience any health consequences during the fire season and are unable to identify the timing of potential adverse effects. Unfortunately, we were unable to collect data during the peak smoke period (October 14–31), as orangutan observations were suspended for one month while the research team focused on fighting fires. As 173 fire hotspots were detected at Tuanan (37% of total) during the period of research suspension, it is entirely possible that health consequences emerged or intensified during that time. Furthermore, the air quality data we obtained from Palangkaraya is probably a conservative estimate of the conditions experienced by orangutans, as more than twice as many fire hotspots were detected at Tuanan than Palangkaraya across the entire fire season.

Nonetheless, our results suggest that smoke-exposure effects persist at least a few months, highlighting the potential for long-term impacts on wildlife populations. Our findings complement a recent study conducted in Singapore, where ecological community acoustic activity was significantly reduced during the 2015 haze produced by Indonesia’s peatland fires, exhibiting only partial recovery 16 weeks after the smoke had dissipated^34^. The impacts on wildlife inhabiting Borneo, where air pollution was 15 times worse than that in Singapore, are likely to be much more severe and could be longer-lasting^34^.

Peatland habitats are integral to the survival of the Critically Endangered Bornean orangutan, whose largest populations inhabit the peat swamp forests of Central Kalimantan^9^. Annual fires in Borneo significantly impact orangutan habitats and populations, burning > 20,000 km^2^ of forest and killing hundreds of orangutans in 2015 alone^35^. Whereas the direct impacts from wildfires are relatively clear, we have little understanding of the indirect effects on surviving wildlife in unburned areas. The unexpected loss of an estimated 93,000 Bornean orangutans from intact forests in Kalimantan between 1999 and 2015 indicates that drivers independent of land-use change are contributing to their population declines^36^. Prolonged and repeated exposure to toxic smoke may have severe health consequences for orangutans and other wildlife, highlighting the urgent need for sustained research into the long-term impacts. Future studies should include non-adults, especially developing foetuses and infants who are likely to be most vulnerable to long-term health effects^37^. We plan to expand this research by including additional physiological indicators of health, which will further elucidate the short- and long-term impacts of wildfire smoke on orangutan health and conservation.

## Electronic supplementary material

Supplementary Table S1
