# Supplementary material for: Wildfire smoke impacts activity and energetics of wild Bornean orangutans
Source: Sci Rep. 2018 May 15;8:7606. doi: 10.1038/s41598-018-25847-1 (PMC5953934; doi:10.1038/s41598-018-25847-1)
Supplement: Supplementary file 1 — Supplementary Table S1 [file 41598_2018_25847_MOESM1_ESM.pdf]

Wildfire smoke impacts activity and energetics of wild Bornean orangutans

*Erb, W.M,[1,2,3] Barrow, E.J.[1,3], Hofner, A.N.[1,3], Utami-Atmoko, S.S.,[3,4,5] Vogel, E.R.[1,2,3]

[1] Department of Anthropology, Rutgers University, New Brunswick, NJ, USA

[2] Center for Human Evolutionary Studies, Rutgers University, New Brunswick, NJ, USA

[3] CORE Borneo, New Brunswick, NJ, USA

[4] Fakultas Biologi, Universitas Nasional, Jakarta, Indonesia

[5] Primate Research Centre, Universitas Nasional, Jakarta, Indonesia

*Corresponding author: erbivorous@gmail.com

Supplementary Table S1. Dataset indicating month, year, individual, smoke period, fruit availability, ketone presence, travel distance, active hours, travel time, rest time, and caloric intake for each nest-to-nest focal animal follow

Mo	Year	ID	Period	FAI	Ket	Dist	Active	Travel	Rest	kCal
3	2015	1	Pre	2.54		1097	12.4	14	19	4899
3	2015	1	Pre	2.54	0	751	10.9	11	29	3821
3	2015	1	Pre	2.54	0	704	12.1	8	15	3685
3	2015	1	Pre	2.54	0	749	12.8	10	14	3604
3	2015	1	Pre	2.54	0	1407	12.4	16	17	4268
5	2015	2	Pre	1.95	0	412	7.7	5	46	606
5	2015	2	Pre	1.95	0	528	9.8	4	30	2693
5	2015	2	Pre	1.95	0	499	9.8	4	38	1169
5	2015	2	Pre	1.95	0	1436	10.2	10	30	1470
6	2015	3	Pre	2.57	0	665	10.6	6	21	3539
6	2015	3	Pre	2.57	0	477	9.1	4	30	2775
6	2015	3	Pre	2.57	0	924	10.5	7	28	2884
6	2015	3	Pre	2.57	0	811	10.4	8	15	2828
7	2015	4	Pre	4.42	0	628	11.1	5	27	1460
7	2015	4	Pre	4.42	0	374	9.8	4	26	1083
7	2015	4	Pre	4.42	0	426	10.4	2	29	1455
7	2015	4	Pre	4.42	0	286	10.9	2	19	1668
7	2015	4	Pre	4.42	0	1162	12.2	7	34	1187
9	2015	4	Smoke	4.48	0	347	11.9	2	61	2634
9	2015	4	Smoke	4.48	0	176	11.4	3	55	861
9	2015	4	Smoke	4.48	0	785	10.5	5	60	2341
9	2015	1	Smoke	4.48	0	1107	11.3	14	43	
9	2015	1	Smoke	4.48	0	168	11.2	1	69	1475
9	2015	1	Smoke	4.48	0	234	10.3	5	66	1680
9	2015	1	Smoke	4.48	1	493	10.8	6	62	1656
9	2015	1	Smoke	4.48	0	999	11.2	8	58	2242
9	2015	3	Smoke	4.48	0	430	10.8	5	57	5194
9	2015	3	Smoke	4.48	0	232	8.8	5	50	3857
9	2015	3	Smoke	4.48	0	979	11.6	9	56	5510
9	2015	4	Smoke	4.48	0	1191	10.9	9	46	3185
9	2015	4	Smoke	4.48	0	858	9.7	9	43	2488
10	2015	2	Smoke	4.69	0	487	11.9	2	49	3029
10	2015	2	Smoke	4.69	1	1561	9	8	42	1975
10	2015	3	Smoke	4.69	0	360	10.5	3	39	3891
10	2015	3	Smoke	4.69	0	686	10.8	4	38	4460
10	2015	2	Smoke	4.69	1	243	10.3	2	26	5260
10	2015	2	Smoke	4.69	1	178	9.4	2	20	5502
10	2015	2	Smoke	4.69	1	308	9.2	3	23	4581
11	2015	2	Post	3.45	1	265	9.7	2	31	3646
11	2015	2	Post	3.45		526	10.4	2	41	4029
11	2015	4	Post	3.45	0	454	10.9	2	56	5153
11	2015	4	Post	3.45	1	190	11.7	1	44	5176
11	2015	4	Post	3.45	0	157	10.4	1	44	3562
11	2015	4	Post	3.45	0	181	11.6	1	47	5202
11	2015	4	Post	3.45	0	342	10.8	1	40	4168
11	2015	2	Post	3.45		452	10.2	3	27	2938
12	2015	2	Post	3.23		326	11.4	3	50	5510
12	2015	2	Post	3.23	0	352	9.8	3	26	3843
12	2015	3	Post	3.23	0	643	10.7	5	37	4042
12	2015	3	Post	3.23	1	143	9.8	2	18	6569
12	2015	3	Post	3.23	1	342	10.9	2	45	5315
12	2015	3	Post	3.23	0	278	11.8	2	31	5440
12	2015	3	Post	3.23	0	418	12.3	3	25	5958
1	2016	2	Post	2.37	1	271	10.4	2	64	2334
